# Water interaction and bond strength to dentin of dye-labelled adhesive as a function of the addition of rhodamine B

**DOI:** 10.1590/1678-775720150447

**Published:** 2016

**Authors:** Linda WANG, Odair BIM, Adolfo Coelho de Oliveira LOPES, Luciana Fávaro FRANCISCONI-DOS-RIOS, Rafael Massunari MAENOSONO, Paulo Henrique Perlatti D’ALPINO, Heitor Marques HONÓRIO, Maria Teresa ATTA

**Affiliations:** 1 - Universidade de São Paulo, Faculdade de Odontologia de Bauru, Departamento de Dentística, Endodontia e Materiais Odontológicos, Bauru, SP, Brasil.; 2 - Universidade de São Paulo, Hospital de Reabilitação de Anomalias Craniofaciais, Divisão de Prótese, Bauru, SP, Brasil.; 3 - Universidade de São Paulo, Faculdade de Odontologia, Departamento de Dentística, São Paulo, SP, Brasil.; 4 - Universidade Anhanguera-Bandeirantes, Grupo de Pesquisa de Biomateriais, São Paulo, SP, Brasil.; 5 - Universidade de São Paulo, Faculdade de Odontologia de Bauru, Departamento de Odontopediatria, Ortodontia e Saúde Coletiva, Bauru, SP, Brasil.

**Keywords:** Fluorescent dyes, Dentin-bonding agents, Water, Absorption, Tensile strength

## Abstract

**Objective:**

This study investigated the effect of the fluorescent dye rhodamine B (RB) for interfacial micromorphology analysis of dental composite restorations on water sorption/solubility (WS/WSL) and microtensile bond strength to dentin (µTBS) of a 3-step total etch and a 2-step self-etch adhesive system.

**Material and Methods:**

The adhesives Adper Scotchbond Multi-Purpose (MP) and Clearfil SE Bond (SE) were mixed with 0.1 mg/mL of RB. For the WS/WSL tests, cured resin disks (5.0 mm in diameter x 0.8 mm thick) were prepared and assigned into four groups (n=10): MP, MP-RB, SE, and SE-RB. For µTBS assessment, extracted human third molars (n=40) had the flat occlusal dentin prepared and assigned into the same experimental groups (n=10). After the bonding and restoration procedures, specimens were sectioned in rectangular beams, stored in water and tested after seven days or after 12 months. The failure mode of fractured specimens was qualitatively evaluated under optical microscope (x40). Data from WS/WSL and µTBS were assessed by one-way and three-way ANOVA, respectively, and Tukey’s test (α=5%).

**Results:**

RB increased the WSL of MP and SE. On the other hand, WS of both MP and SE was not affected by the addition of RB. No significance in µTBS between MP and MP-RB for seven days or one year was observed, whereas for SE a decrease in the µTBS means occurred in both storage times.

**Conclusions:**

RB should be incorporated into non-simplified DBSs with caution, as it can interfere with their physical-mechanical properties, leading to a possible misinterpretation of bonded interface.

## INTRODUCTION

For three decades, dental researchers have incorporated fluorescent dyes into adhesive systems to perform *in vitro* ultra-morphological assessment of the tooth-adhesive interface via confocal laser scanning microscopy (CLSM)^3,18,26^. The labeling of dental adhesives refers to a simple mixing process between the fluid resin and a fluorescent dye, like rhodamine or fluorescein^3,5^. These dyes have not been covalently attached to crosslinking monomers, but simply mixed with fluid resins. During the adhesive polymerization, the dye molecules get entrapped into the polymer network, labeling it. Considering that fluorescent dyes shall fluoresce under suitable laser excitation^19^, the path of a labeled adhesive within the bond interface can be easily highlighted in dentin-adhesive specimens prepared for laser scanning microscopy^3,24^.

Rhodamine B (RB) is one of the most commonly utilized dyes for adhesive labeling^3^. It presents excellent photophysical properties such as high molar absorptivity and quantum yield^2^. In other words, RB efficiently absorbs light energy (peak absorption wavelength is usually in the green color region) and reemits most of it as fluorescence into longer, lower energy wavelengths. Besides, RB powder is readily soluble in water and organic solvents, such as ethanol^2^, which is frequently found in the composition of the simplified dental adhesives^21,27^.

Though CLSM is considered a powerful high-resolution and non-destructive method for qualitative investigations on dental bonding, there should be awareness of potential factors limiting the reliability of the bond integrity analysis. A few studies have addressed concerns with the lack of standardization on the concentration of RB and other dyes for adhesive labeling^2,3^. The amount of RB in the dentin bonding systems (DBSs) must be suitable for the CLSM analysis and, on the other hand, RB must not interfere with the mechanisms of dental bonding or hybridization. Otherwise, it could result in corrupted morphological patterns and misinterpretation of the tooth-adhesive interface^3,25^. Regarding this matter, the impact of the addition of RB to a simplified total-etch DBS was previously investigated^2^. A RB concentration of 0.16 mg/mL was established for that specific DBS as a safe boundary for its association in terms of bond strength and monomer conversion. The same RB concentration was adopted for adhesive labeling in other investigations^4,14^. However, possible effects of the addition of RB to non-simplified DBSs have not been addressed in the literature yet. Current DBSs can differ from each other in functional monomers, pH, solvents, and mode of interaction with the moist dentin substrate^17,21^. The 3-step etch-and-rinse and the 2-step self-etching systems have been considered the gold standard adhesives, as these materials present improved laboratorial and clinical performances^1,13^. With regard to the adhesive labeling, these non-simplified adhesives present higher viscosity and are very hydrophobic in comparison with the simplified ones, thus limiting proper dissolution of RB.

The purpose of this study was to evaluate the effect of addition of RB to two non-simplified commercial DBSs on water sorption/solubility and microtensile bond strength to dentin. Drawing upon two hypotheses, this study attempts to investigate the effects of DBS labeling with RB on water sorption, solubility, and bond strength to dentin of two commercial systems (a conventional, 3-step adhesive and a 2-step, self-etching adhesive). The hypotheses tested were as follows: (1) the RB affects the water sorption and the solubility of the adhesive systems tested; (2) the RB influences the bond strength to dentin, irrespective of the evaluation time (seven days or 12 months).

## MATERIAL AND METHODS

**Figure 1:**
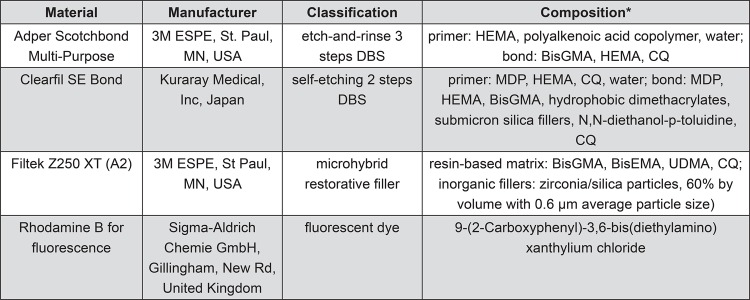
Information about the materials used in this study

The main materials used in this study are described in Figure 1.

*HEMA: 2-Hydroxyethyl methacrylate

BisGMA: Bisfenol diglycidyl dimethacrylate

CQ: camphorquinone

MDP: 10-methacryloyloxydecyl-dihydrogen phosphate

BisEMA:ethoxylated bisphenol-A dimethacrylate

UDMA: Urethane dimethacrylate

### Adhesive labeling with rhodamine B

**Figure 2:**
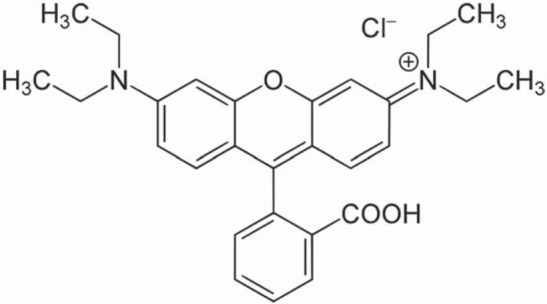
Chemical structure of rhodamine B

Rhodamine B (Rhodamine B^®^, Sigma-Aldrich Chemie GmbH, Gillingham, New Rd, UK) was used according to the manufacturer’s instructions (no further purification was performed) and its chemical formula is indicated in Figure 2. Rhodamine B powder (≈1.0 mg) was weighed on an analytical balance (GR-202, A&D Engineering, Inc., San Jose, CA, USA) inside a small Eppendorf tube. This procedure was performed in duplicate. The two tubes were wrapped in aluminum folium and the adhesive component of each DBS (≈10 mL) was then transferred to the corresponding one. Each tube was carefully adapted to a dental mixer and vigorously mixed for 40 s, in order to dissolve the RB in the resin. After mixing, no RB clusters could be detected in the labeled adhesives with the naked eye. The final concentration of RB in each experimental DBS was approximately 0.10 mg/mL, just about the same concentration as previously proposed^2^.

### Water sorption (WS) and water solubility (WSL) tests

Ten disk-shaped specimens of each tested adhesive were prepared for the following groups: MP (control adhesive), MP-RB (with 0.10 mg/mL rhodamine), SE (control adhesive), and SE-RB (with 0.10 mg/mL rhodamine). Control and RB-labeled adhesives were directly placed into a stainless steel mold (≈5.0 mm in diameter and 0.8 mm thick) until it was completely filled. A polyester strip was then placed over the fluid resin and compressed with a glass slide^8^. The experimental groups were light-cured with a light emitting diode curing unit at 1,200 mW/cm^2^ for 30 s (Radii-cal^®^, SDI Limited, Bayswater, VIC, Australia). The adhesive disks were subsequently removed from the mold and excess flash was cut off using a scalpel blade. The mean thickness (h) of each specimen was obtained by measuring three equidistant points on its base with a digital electronic caliper (Mitutoyo Corporation, Tokyo, Japan), and the volume (V) of the specimen was calculated by *V*=*h*×(2.5)^2^×3.14. Water sorption and solubility tests were based on the 4049 ISO standard with the exception of the specimen size. The adhesive disks were then individually stored in a desiccator (37°C) containing silica gel. Each disk was repeatedly weighted in a calibrated analytical balance (TP-214, Denver Instrument, Denver, CO, USA) in 24-hour intervals, until a constant mass was obtained (m_1_). Subsequently, the disks were immersed in deionized water in individual vials. During seven days and within 24-hour intervals, the specimens were removed from water, carefully blotted with an absorbent tissue paper, weighted and returned to water until a constant mass was obtained (m_2_). After this, each specimen was submitted to a new desiccation cycle until a constant mass was obtained (m_3_). The values of WS and WSL were calculated by equations 1 and 2 respectively:


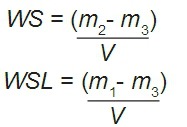


Also, net water uptake, which represents the sum of water sorption and solubility in percentage, was calculated for each condition.

### Microtensile bond strength (µTBS) test and CLSM of dentin-adhesive interfaces

**Figure 3:**
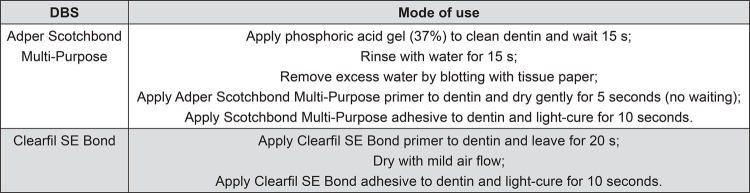
Instructions for use of DBSs in dentin for direct composite restorations

Extracted sound human third molars, obtained by donation from patients who signed an informed consent beforehand, were included in this study. Ethical protocol was approved by the Ethics Committee for Human Studies (process number 118/2011). The occlusal third of the forty molar crows was cut by a diamond disk (Extec Corp, Enfield, CT, USA) using a low-speed saw cutting machine (Isomet, Buehler Ltd, Lake Bluff, IL, USA), exposing flat deep dentin surfaces. Subsequently, dentin surfaces were submitted to a water-cooled 600-grit SiC paper abrasion (Buehler Ltd, Lake Bluff, IL, USA) to create standardized surfaces. Then, the specimens were assigned into 4 groups, regarding the bonding protocol as previously described: MP and SE controls (no dye), and RB labeled groups (n=10). The DBSs were then applied to the dentin surfaces according to the manufacturers’ instructions in Figure 3. Photoactivation was performed for 10 s using the same LED light (Radii-cal^®^, SDI Limited, Bayswater, VIC, Australia). Composite buildups (3.0 mm in height) were incrementally constructed with a resin composite (Filtek Z250, 3M ESPE, St. Paul, MN, USA). After the bonding procedures, the crowns were buccolingually cut into ≈0.9-mm thick slices parallel to the tooth’s long axis, using the same low-speed saw and diamond disk. At that stage, one slice of each crown from the groups MP-RB and SE-RB was randomly selected to be analyzed via CLSM, using diode laser scanning with a 532 nm laser excitation wavelength (Leica TCS SPE, Leica Microsystems CMS, Mannheim, Germany).

Then, the remaining slices (all groups) were mesiodistally sectioned into rectangular beams with a cross-sectional area of 0.8 mm^2^ approximately. The dentin-resin specimens were stored in deionized water at 37°C. Half of them were tested after 7-day storage and the other half after 12 months of storage. Beams were individually fixed to a custom-made testing jig (Bencor Multi T’s like device) with cyanoacrylate glue (Super Bonder Flex Gel Loctite^®^; Henkel Ltda., São Paulo, SP, Brazil) and subjected to tensile load (50 kgf load cell) at a crosshead speed of 0.5 mm/min until bond failure (Instron, Model 3342, Norwood, MA, USA). In this experiment, bond strength to dentin involved two factors: DBSs (MP or SE) under different conditions (neat adhesives or labelled with RB) and different storage times (7-day or 12 month evaluation), all in two levels.

### Failure mode analysis

Fractured dentin-resin interfaces were analyzed under a digital microscope with 40x magnification (Dino-Lite Digital Microscope^®^, AnMo Electronics Corp., New Taipei City, San-Chung District, Taiwan). Failure modes were classified as adhesive failure (A), mixed failure (M), cohesive failure in resin composite (CC), and cohesive failure in dentin (CD).

### Statistical analysis

Data were analyzed with Statistica statistical package 11.0 (Tulsa, OK, USA). The assumptions of equality of variances and normal distribution of errors for all the variables were checked (Kolmogorov-Smirnov). As the assumptions were satisfied, one-way and three-way ANOVA regarding WS/WSL and µTBS, and Tukey’s test was carried out for statistical comparisons (α=0.05).

## RESULTS

**Figure 4:**
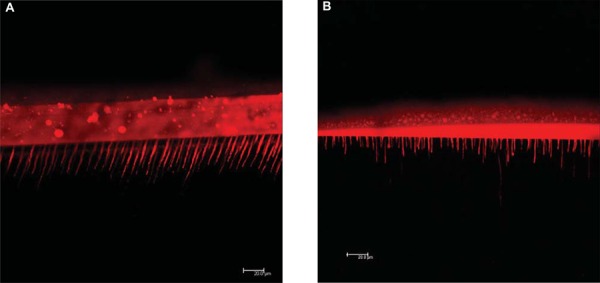
A- Confocal laser scanning microscopy. Dentin-adhesive interface with Adper Scotchbond Multi-Purpose labeled with rhodamine B (0.10 mg/mL in the adhesive component). 532 nm laser excitation wavelength, 10% laser range, x40/1.15 NA in oil immersion. B- Confocal laser scanning microscopy. Dentin-adhesive interface with Clearfil SE Bond labeled with rhodamine B (0.10 mg/mL in the adhesive component). 532 nm laser excitation wavelength, 10% laser range, x40/1.15 NA in oil immersion

Representative CLSM photomicrographs of dentin-MP and dentin-SE interfaces are shown in Figures 4A and 4B respectively. Adding 0.10 mg/mL of RB to the adhesive systems tested produced intense fluorescence in the labeled samples during the laser scanning microscopy. Regarding the interfacial interlocking patterns registered in the photomicrographs, specimens from the group MP-RB presented more and longer resin tags than the ones in the SE-RB group.

The distribution of dye-labeled adhesive throughout demineralized dentin with great resolution and the quality of the hybrid layer imaged was greatly enhanced using the proposed fluorescent dye concentration. The hybrid and adhesive layer thicknesses (intense red) are also clearly discernible as well as the characteristics of resin tags. However, comparing both figures, SE produced shorter tags than shown by MP. Also, the hybrid layer seems to be better identified for MP.

WS and WSL results are shown in Table 1. Rhodamine B caused an increase in WSL for MP and SE. On the other hand, WS of MP or SE was not affected by the addition of the dye. The net water uptake for both adhesives was determined to be similar, irrespective of the presence of the RB or not.

**Table 1:** Water sorption (WS) and solubility (WSB) in µg/mm3 of neat and RB-labelled adhesives

	Water sorption		Solubility		Net water uptake (%)**
	(µg/mm^3^)	(%*)	(µg/mm^3^)	(%*)	
MP	90.7 (13.0)^a^	9.07	14.4 (8.3)^b^	1.44	10.51
MP-RB	109.8 (5.6)^a^	10.98	25.9 (9.3)^a^	2.59	13.57
SE	96.4 (8.5)^a^	9.64	-7.0(4.0)^c^	-0.70	8.94
SE-RB	96.4 (4.9)^a^	9.64	19.9 (9.3)^a^	1.99	11.63

Values are mean (standard deviation), n=10, µg/mm^3^. Water sorption is given in absolute terms (µg/mm^3^) and in relative terms (%) to provide comparisons to literature values which include both expressions.

Different small letters in column: significant (p<0.05).

*90.7 µg/mm^3^ = 0.0907 mg/mm^3^ ×100 = 9.07 mg/100 mm^3^ = 9.07 %.

** Net water uptake is the sum of water sorption and solubility (%).

**Figure 5:**
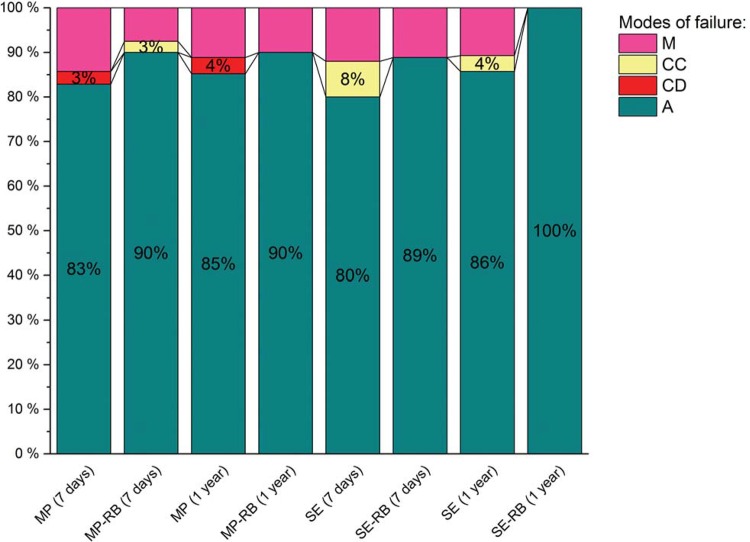
Failure mode distribution. (M) mixed failure, (CC) cohesive failure in composite resin, (CD) cohesive failure in dentin, and (A) adhesive failure

Table 2 presents the results of the µTBS test (in MPa) and comparisons among the experimental groups. By adding RB to Adper Scotchbond Multi-Purpose groups, no influence in terms of bond strength were observed at 7-day analysis, with or without RB associated. After 12 months, no differences between them were found again; however, a significant decrease was shown of BS for both groups in comparison with their initial values. To Clearfil SE Bond groups, RB decreased the bond strength to dentin, in both evaluation times. In the DBSs control groups (MP and SE), no difference on bond strength was observed in both testing times. All groups were associated predominantly with adhesive failures, as showed in Figure 5.

**Table 2:** Mean (MPa) and standard deviation (SD) values of bond strength to dentin of neat and RB-labelled adhesives

Material	Group	Time	Mean (SD)
Adper Scotchbond Multi-Purpose	MP	7 days	39.58 (10.21)^a^
	MP-RB	7 days	35.13 (9.81)^a^
	MP	12 months	21.00 (5.23)^bc^
	MP-RB	12 months	9.73 (3.44)^bd^
Clearfil SE Bond	SE	7 days	43.60 (18.10)^a^
	SE-RB	7 days	20.27 (6.38)^bc^
	SE	12 months	30.26 (9.75)^ac^
	SE-RB	12 months	5.21 (6.48)^d^

n=10 - Different letters mean significant statistically differences (p<0.05)

## DISCUSSION

Dental bonding through hybridization depends primarily on physicochemical interactions between the moist dentin substrate and the resin-based DBSs^11,12^. Besides good resin infiltration, adequate polymerization is fundamental for the immediate mechanical performance of the dentin-resin bond interface^22,23^. Modifying the original composition of a DBS, by adding a fluorescent dye, such as RB, for example, can ultimately interfere with dental bonding and limit the bond integrity analysis by CLSM. According to the present study, the RB concentration of 0.10 mg/mL in the adhesives permitted the detection of the resin distribution in dentin-MP and dentin-SE interfaces by CLSM (Figures 4A and 4B), but it has also negatively affected some resin properties.

In an attempt to avoid errors in the interpretation of the results, it is relevant to reinforce that rhodamine B did not covalently attach to crosslinking monomers, being inert. During the adhesive polymerization, the dye molecules are entrapped into the polymer network, labeling it. Therefore a previous analysis of degree of conversion (unpublished data) was performed regarding the addition of 0.10 mg/mL of RB, which did not interfere with this property for both the systems.

Dental literature shows that WS/WSL and µTBS tests are commonly employed to compare characteristics between distinct DBSs, as well as to predict the quality of dental bonding, and even the long-term clinical performance of such resin-based materials^6,16^. Though RB is not intended for any clinical use under the approach outlined in this paper, the interlocking pattern, which the dye highlights through CLSM observations, is expected to be similar to the bonding patterns normally obtained in the clinical situation – otherwise the morphological assessment could be dubious.

Regarding the response variables addressed in this study, the results indicate that the effect of RB (0.10 mg/mL) on WS/WSL and µTBS seems to be material-dependent.

Control groups of the adhesives MP and SE showed similar WS values. These DBSs pertain to distinct categories (etch-and rinse and self-etching respectively), but both are non-simplified systems presenting in separate bottles the same hydrophobic cross-linker resin component, which is known for providing higher polymer stability under wet conditions^15^. Previous studies have indicated that increasing WS of DBSs may precede degradation processes, which impact the long-term stability of the polymer matrix in wet environments, thus flawing the quality of the dentin-resin interlocking^9,11^. Based on the present data, RB did not cause any negative effect to WS for any of the tested conditions. However, the addition of RB in the SE adhesive caused an increase in WSL. Based on the analysis of net water uptake, which represents the sum of WS and WSL, it indicates a balanced performance between all tested conditions, which calls for attention to their interpretation. In terms of bond strength, the 3-step etch-and-rinse DBS was less affected from the addition of this dye than the 2-step self-etching one. The elucidation of the significant decrease in µTBS in the groups SE-RB (7-day and 12-month tests) would demand further investigations regarding other polymer properties, and also with the mode of interaction of mild self-etching adhesives with dentin. Their bonding mechanism to dentin relies primarily on the capacity of its self-etching functional monomers to remove minerals of the moist dentin matrix, enabling concomitant resin infiltration and interfacial interlocking^10,29^. Furthermore, the SE system presents chelating functional monomers in its composition, known for fomenting the occurrence of chemical bonding with residual hydroxyapatite^28,30^. Considering this, it could be beneficial to investigate if RB can affect the pH of the system SE and impair its self-etching bonding mechanism.

Figures 4A and 4B show a very intense fluorescence sign of RB in the bonding interfaces. The concentration of RB seems to be higher than that necessary for a suitable CLSM analysis, and the intense fluorescence can difficult the distinction of micromorphological structures. This can possibly be the reason why the hybrid layer of SE in Figure 4A is not evident. It could be advantageous to investigate some characteristics of RB photophysics, when the dye is dispersed in different cured adhesives. Fluorescence emission is a phenomenon influenced by a series of factors, such as polarity, viscosity and pH of the microenvironment, and by the concentration of fluorescent dye itself^7,20^. Therefore, a preliminary evaluation of the photophysical behavior of fluorescent dyes in different polymer-based materials could provide valuable information, aiming to determine suitable RB concentrations for the bond analysis by CLSM.

## CONCLUSIONS

Rhodamine B (0.10 mg/mL), as a fluorescent dye for the micromorphologycal analysis, can negatively affect the WSL of both systems and the µTBS of Clearfil SE Bond. The dye should be incorporated to non-simplified DBSs with caution, as it can interfere with their physical-mechanical properties, leading to bias in the bond integrity analysis, especially for overtime bond strength analysis.
